# Quantitative Effect of a CNV on a Morphological Trait in Chickens

**DOI:** 10.1371/journal.pone.0118706

**Published:** 2015-03-13

**Authors:** Céline Moro, Raphaël Cornette, Agathe Vieaud, Nicolas Bruneau, David Gourichon, Bertrand Bed’hom, Michèle Tixier-Boichard

**Affiliations:** 1 Institut National de la Recherche Agronomique, AgroParisTech, UMR1313 Animal Genetics and Integrative Biology, Jouy-en-Josas, France; 2 Museum National d’Histoire naturelle, UMR 7205 Institut de Systématique, Évolution, Biodiversité, 57 rue Cuvier, Paris, France,; 3 Institut National de la Recherche Agronomique, UE1295 Pôle d’Expérimentation Avicole de Tours, Nouzilly, France; University of Maryland, UNITED STATES

## Abstract

Copy Number Variation has been associated with morphological traits, developmental defects or disease susceptibility. The autosomal dominant Pea-comb mutation in chickens is due to the massive amplification of a CNV in intron 1 of *SOX5* and provides a unique opportunity to assess the effect of variation in the number of repeats on quantitative traits such as comb size and comb mass in Pea-comb chickens. The quantitative variation of comb size was estimated by 2D morphometry and the number of repeats (RQ) was estimated by qPCR, in a total of 178 chickens from 3 experimental lines, two of them showing segregation for the Pea-comb mutation. This study included only Pea-comb chickens. Analysis of variance showed highly significant effects of line and sex on comb measurements. Adult body weight (BW) and RQ were handled as covariates. BW significantly influenced comb mass but not comb size. RQ values significantly influenced comb size, and the linear regression coefficient was highest for heterozygous carriers: the higher the number of repeats, the smaller the comb size. A similar trend was observed for comb mass. The CNV contributed to 3.4% of the phenotypic variance of comb size in heterozygous carriers of the CNV, an order of magnitude frequently encountered for QTLs. Surprisingly, there was no such relationship between RQ values and comb size in the homozygous line. It may be concluded that heterozygosity for a CNV in a non-coding region may contribute to phenotypic plasticity.

## Introduction

Since the onset of whole genome sequencing projects, Copy Number Variation, i.e variation due to a change in the number of copies of DNA segments ranging from 1 kb to several Mb [1], has attracted a growing interest. The genomic architecture of CNV has been characterized for several species of domestic animals, including chickens [2, 3]. CNVs have been shown to modify gene expression, either because of dosage effect for genes within the CNV, or because of structural changes disturbing expression of neighboring genes [4]. A common hypothesis today is that CNVs may have an important impact on phenotypes, as shown for diseases in humans [5]. Whereas quantitative effects of CNVs have been described on gene expression [4], the possibility that CNVs may affect quantitative variation of continuous traits has been documented in only a few instances [6, 7]. Such an effect may be of high importance for animal performance as well as for the general understanding of the genetic regulation of quantitative traits.

The autosomal dominant Pea-comb mutation in chickens is one such example where a CNV, located in intron 1 of a development gene, *SOX5*, has been found to be associated with a change in comb size and morphology, due to an ectopic expression of *SOX5* during comb morphogenesis [8]. As compared to the wild-type comb (also called single-comb), the Pea-comb mutation reduces the size and greatly modifies the shape of the comb. In mutant birds, a segment of 3.2 kb was found to be repeated 20 to 40 times, 16kb upstream from the first non-coding exon of *SOX5*, whereas this segment was only repeated twice in wild-type animals. Thus, the *SOX5*-CNV in Pea-comb birds results from an expansion of a pre-existing duplication and not from a de novo event. Furthermore, it involves only a non-coding region. A marked variation in the number of repeats was observed between Pea-comb birds, and it was not possible to distinguish homozygous from heterozygous carriers on the basis of the number of repeats [8]. Furthermore, a large QTL region controlling female comb size and overlapping the *SOX5* locus was detected in a large intercross between red jungle fowl and a domestic line of chickens [9]. More recently, the Pea-comb mutation was used as a model to establish the importance of the Sonic hedgehog-signalling pathway in comb morphogenesis [10]. Since comb size and comb mass are quantitative traits, the Pea-comb mutation provides a unique opportunity to assess the effect of variation in the number of repeats on the quantitative value of a trait modified by a CNV.

The aim of the present study was to assess the relationship between the variable number of repeats of the SOX5-CNV, estimated by qPCR, and the quantitative variation of comb mass and comb size, estimated by 2D morphometry, in a set of 3 experimental lines of chickens carrying the Pea-comb mutation.

## Material and Methods

### Animals

Three experimental lines of egg-type chickens were used. The CH1 line is fixed for the Pea-comb phenotype: all chickens in this line are homozygous carriers of the Pea-comb mutation. The WL-DJ line is a White Leghorn line selected for multiple ovulations, and the NOE line is a resource population segregating for several mutations. The WL-DJ and the NOE lines are segregating for the Pea-comb mutation. In these two lines, the present study only considered the chickens exhibiting the Pea-comb phenotype, which could be either homozygous or heterozygous carriers of the mutation. Heterozygosity status could not be fully determined since there is no SNP diagnostic of the Pea-comb allele. In the WL-DJ and the NOE lines, those animals having one wild-type parent, or at least one wild-type offspring, may be scored as heterozygous carrier of Pea-comb. A few animals were scored homozygous because they produced at least 7 pea-comb offspring when mated with a wild-type animal. All other animals studied for these two lines had an undetermined (ND) carrier status for Pea-comb but showed the Pea-comb phenotype (Table 1).

**Table 1:** Number of animals recorded per line, sex, trait and Pea-comb genotype.

	CH1 line		WL-DJ line		NOE lin			Total
Sex	male	female	male	female	male	female		
Comb size	24	39	17	39	21	38		178
Comb mass	20	28	17	35	19	31		150
Body weight	23	39	17	39	21	38		177
RQ values	20	37	11	36	16	37		157
Pea-comb genotype according to pedigree data								
Homozygous	24	39	0	3	2	3	71	
Heterozygous	0	0	10	22	12	21	65	
Undetermined carrier status (ND)	0	0	7	14	7	14	42	

Adult females were housed in individual cages for precise egg production recording and adult males were housed in a different room, being 1 or 2 in a cage. The mean ambient temperature was set to 21°C. Light duration was 14 hours a day for females and 12 hours a day for males. Animals were fed *ad libitum* with a layer diet for females, and a maintenance diet for males. The total number of animals included in this study was 178, with 62 males and 116 females (Table 1).

### Ethics statement

All the chickens from the INRA experimental farm PEAT were produced, fed and sacrificed in 2011, according to French regulations for animal care, which, at that time, did not require the approval by an ethical committee, but an administrative authorization of the facility and the researchers. The farm was, and still is, registered by the ministry of Agriculture with license number B37–175–1 for animal experimentation. The experiment was done under authorization 37–002 delivered to D. Gourichon, authorization 2369 delivered to M. Tixier-Boichard and authorization 78–145 delivered to A. Vieaud. Animal procedures were approved by the Departmental Direction of Veterinary Services of Indre-et-Loire. Before tissue sampling, animals were sacrificed by electronarcosis followed by decapitation.

### Measurements of comb size and comb mass

Adult animals were weighed at the age of 300 days. Blood sampling was performed as part of the routine monitoring of the lines to get 1 ml of whole blood for DNA extraction and storage. Pictures of the head of live chickens were taken from each side. This was performed with a Nikon Coolpix L110 set in Macro mode with a 15 X Nikkor lens (Optical Zoom Wide VR 5.0–75.0 mm 1:3.5–5.4) with a 6X magnifying power and a distance of 27 cm between objective and target. At the end of the reproduction period, i.e. at the age of 390 days, animals were sacrificed and individual combs were dissected, cleaned if necessary, and weighed at the nearest 0.1 g. Since some birds had died in the meantime between comb size measurement and comb mass determination, or were still needed for other experiments, the dataset for comb mass was reduced to 150 observations (Table 1).

**Fig 1:**
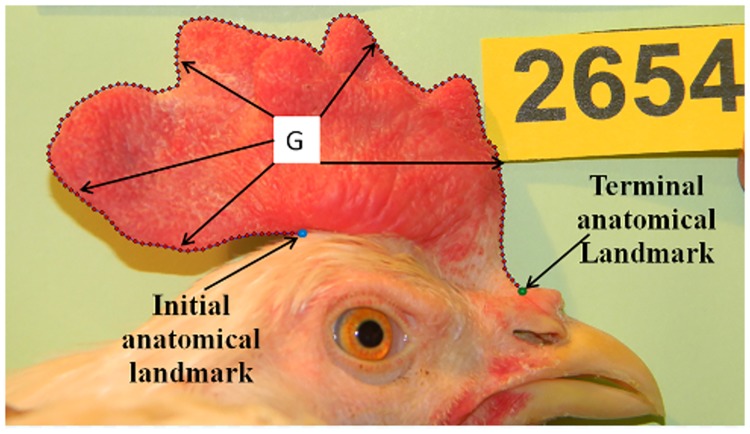
Landmark recording on the picture of the Pea-comb from an adult male (animal #2654); G represents the center of gravity.

Image analysis was performed on one picture per bird, corresponding to the one where the comb was the most straight, in order to maximize the surface available for the analysis. Geometric morphometry relies on the Thin Plate Splines approach (TPS) in order to quantify the shape of the comb, and to separate ‘size’ from ‘conformation’ in a second step. The surface of the comb was first outlined on each picture by defining 150 anatomical landmarks between the proximal and the distal ends of the comb (Fig. 1), using the tpsDig2 software.

Then, images were analyzed by the Procruste process following 3 steps: (i) translation of all objects to superpose them at their center of gravity (ii) normalization to a reference scale for each object (iii) rotation of each object to minimize the distances between its landmarks and the landmarks of the consensus object. This process allows the analysis of size variation independently from shape variation. This is particularly important for Pea-comb, which has a more irregular shape than the wild-type comb, so that a simple measurement of width and height would not be reliable. Landmark coordinates (also called Procruste residuals) can be used to compare the conformation of each object [11]. Finally, the tpsRelw software was used to calculate the centroid size (CS):

$$CS=\sqrt{\sum_{i=1}^{150}{\left(L_{i}-G\right)}^{2}}$$

where G is the center of gravity for each comb picture and L_i_ the ith landmark. CS was used as a measure of comb size in an arbitrary unit, uncorrelated to conformation, for the remaining of the study.

### Molecular analysis

Genomic DNA was obtained from 20 μl whole blood, and extracted with a fast method providing high quality DNA in birds, as previously described [12]. Final quality and quantity of DNA extracts were determined with Nanodrop ND-1000 Spectrophotometer.

The qPCR procedure previously developed for the identification of the Pea-comb mutation [8] was used with the same primers and experimental conditions, including the rps24 gene as an internal control. A fragment of 110 bp was amplified within the repeated region upstream of *SOX5*, and a fragment of 75 bp was amplified for rps24. A DNA sample from a brown Leghorn bird was used as a single-comb control of *SOX5* CNV. The qPCR procedure was performed with an ABI 7900 HT on the ICE facility for microgenomics at INRA, Jouy-en-Josas. The following formula was used to quantify the number of repeats, RQ:

$$RQ=2^{-(\Delta Ct_{repeat}-\Delta Ct_{rps24})}$$

### Statistical analysis

Elementary statistics were first calculated for comb size, comb mass, body weight and RQ. The individual data for these traits are provided in S1 File. The distribution of RQ values was plotted according to line, genotype and sex. For each line by sex combination, phenotypic correlations were calculated between comb size, comb mass, body weight and RQ values.

For both comb size and comb mass, we fitted models including the fixed effects of line, sex, and the line*sex interaction, as well as body weight and RQ as covariates, to three independent subsets of the data: the full dataset, a subset containing only the WL-DJ and NOE lines, and a subset made up only of the heterozygous carriers of the Pea-comb mutation. We also considered a set of reduced models where the RQ covariate was removed from each of the full models described above; this allowed the calculation of the amount of variance explained by RQ in each case by comparing the proportion of the total variance explained by the full and reduced models. Finally, we also considered a model fit on the CH1 observations alone, including only a fixed effect for sex and the two covariates RQ and body weight.

The GLM procedure of SAS was used for each analysis of variance and the CORR procedure of SAS was used to calculate the phenotypic correlations.

## Results

### Phenotypic variation of comb size and mass

**Fig 2:**
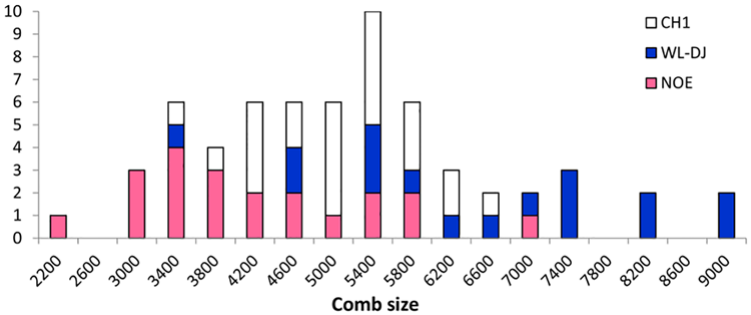
Frequency distribution of comb size measured in arbitrary units for males of each experimental line (CH1, WL-DJ and NOE).

**Fig 3:**
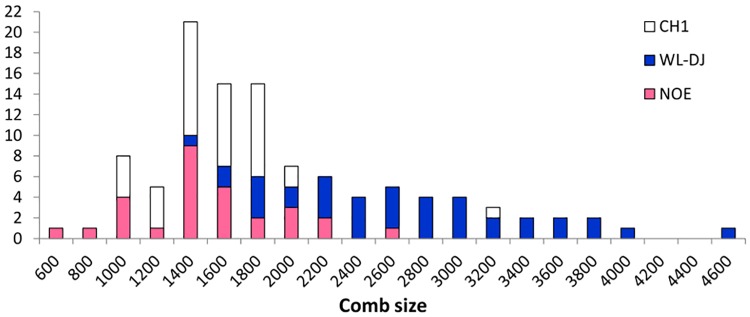
Frequency distribution of comb size measured in arbitrary units for females of each experimental line (CH1, WL-DJ and NOE).

In males, comb size varied from 2399 to 9134 units (Fig. 2) and comb mass from 3 to 40.4g (S1 Table). In females, comb size varied from 636 to 4643 (Fig. 3) and comb mass from 0.2 to 5.9g (S1 Table). The coefficients of variation (CV) tended to be higher for comb size in females (24% to 29% according to lines) than in males (15% to 26% according to lines). Comb mass showed much larger CV than comb size, with values ranging from 34% to 54% in males, and from 31 to 55% in females. The CH1 line showed the lowest CV values. In comparison, CV values were much lower for adult body weight, ranging from 8% to 17% in males, and 9% to 20% in females.

### Phenotypic variation of the number of repeats (RQ)

Among the 178 individuals with data for comb size, only 157 had a valid qPCR result. There were 15 males and 6 females with no qPCR results. They were distributed across lines (6 CH1, 9 WL-DJ, and 6 NOE) and across Pea-comb genotypes (6 homozygous, 7 heterozygous, 8 undetermined). The phenotypic standard deviation (σ_p_) and the CV of RQ were particularly low in males of the WL-DJ line (7%) but reached 38% in females of this line (Table 2). The CV of RQ varied from 16% to 20.5% according to sex in the CH1 line, and from 40 to 43% in the NOE line (Table 2). The WL-DJ line generally exhibited lower values of RQ as compared to the NOE line, particularly in males (Table 2).

**Table 2:** Phenotypic means and standard deviations (in parenthesis) for comb size, comb mass, body weight and RQ values, according to line, sex and Pea-Comb genotype (Ho = homozygous; Het = heterozygous; ND = undetermined carrier status).

Line	CH1	WL-DJ	WL-DJ	WL-DJ	NOE	NOE	NOE
Pea-comb genotype	Ho	Ho	Het	ND	Ho	Het	ND
Comb size of males	5302 (817)	-	7246 (1668)	6014 (1173)	3396 (135)	4457 (1306)	4326 (841)
Comb size of females	1629 (393)	2161 (610)	2649 (653)	3050 (835)	1353 (272)	1744 (484)	1321 (267)
Comb mass (g) of males	13.98 (4.74)	-	28.54 (10.85)	18.67 (8.07)	4.50 (0.85)	9.86 (5.78)	8.91 (3.35)
Comb mass (g) of females	0.75 (0.23)	1.90 (1.22)	2.53 (1.10)	3.26 (1.46)	0.57 (0.23)	0.74 (0.44)	0.58 (0.27)
Body weight (g) of males	2646 (214)	-	2220 (416)	2254 (398)	3640 (219)	3058 (548)	3258 (573)
Body weight (g) of females	1977 (179)	1561 (312)	1744 (359)	1937 (338)	2559 (331)	2339 (412)	2266 (274)
RQ values of males	48.0 (9.87)	-	24.4 (1.74)	23.4 (1.97)	55.9 (5.48)	36.6 (14.5)	57.5 (19.7)
RQ values of females	44.2 (7.17)	42.0 (16.2)	22.4 (3.19)	30.5 (12.5)	44.3 (8.29)	25.9 (8.51)	42.5 (16.7)

**Fig 4:**
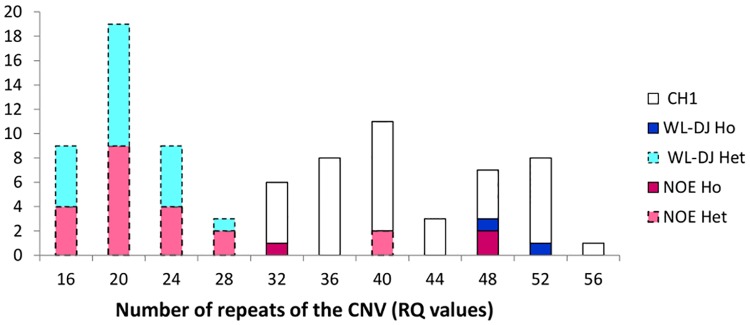
Frequency distribution of the number of repeats (RQ values) for the *SOX5*-CNV in females for each experimental line (CH1, WL-DJ and NOE) according to genotype for the Pea-Comb genotype (Het = heterozygous; Ho = homozygous). All animals of the CH1 line are homozygous.

**Fig 5:**
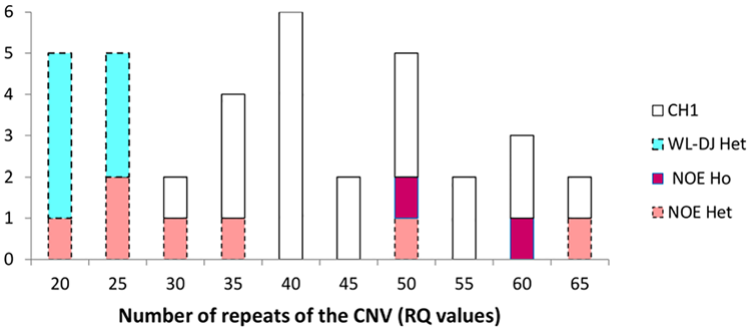
Frequency distribution of the number of repeats (RQ values) for the *SOX5*-CNV in males for each experimental line (CH1, WL-DJ and NOE) according to genotype for the Pea-Comb genotype (Het = heterozygous; Ho = homozygous). All animals of the CH1 line are homozygous.

In females, RQ values for homozygous carriers of Pea-comb from all lines were always higher than 32, whereas the RQ values of heterozygous carriers of Pea-comb in lines WL-DJ and NOE were lower than 30 except for two heterozygous NOE females which exhibited a higher RQ value (Fig. 4). Thus, it would be possible to consider that all ND females showing a RQ value below 30 units were heterozygous for Pea-comb, which would represent 5 females of the NOE line and 10 of the WL-DJ line. In males, however, the RQ values of heterozygous males greatly overlapped with RQ values of homozygous males in the NOE line, and there was no homozygous male in the WL-DJ line, so it was not possible to suggest a Pea-comb genotype for the ND males (Fig. 5).

The phenotypic correlations between comb size and comb mass were high and significant, but generally lower in females as compared to males (Table 3). The phenotypic correlations between body weight and comb mass were moderately positive and significant for females of all lines, but showed quite variable values for males depending on the line, with a positive correlation in the WL-DJ line but a negative one in the CH1 line, and no relationship in the NOE line. Correlations between comb size and body weight were lower than correlations between comb mass and body weight: they were not significant for males and moderately significant for females in the CH1 and WL-DJ lines. Correlations between RQ values and comb size, or comb mass, were significantly negative in the NOE line for both sexes, they were not significant in the other lines, except in males of the CH1 line where a positive correlation was found between RQ and comb mass with a low significance level (p<0.10).

**Table 3:** Phenotypic correlations between comb size (CS), comb mass (CM), body weight (BW), and the number of CNV repeats (RQ), according to line and sex.

	CH1	WL-DJ	NOE
CS-CM in males	0.92 (p<0.0001)	0.85 (p<0.0001)	0.96 (p<0.0001)
CS-CM in females	0.61 (p = 0.0006)	0.82 (p<0.0001)	0.75 (p<0.0001)
CS-BW in males	-0.30 (p = 0.17)	0.31 (p = 0.23)	-0.13 (p = 0.58)
CS-BW in females	0.46 (p = 0.003)	0.45 (p = 0.0039)	-0.004 (p = 0.98)
CM-BW in males	-0.42 (p = 0.073)	0.46 (p = 0.066)	-0.08 (p = 0.75)
CM-BW in females	0.51 (p = 0.0048)	0.57 (p = 0.0004)	0.58 (p = 0.0006)
CS-RQ in males	0.28 (p = 0.23)	0.008 (p = 0.98)	-0.50 (p = 0.047)
CS-RQ in females	-0.02 (p = 0.93)	-0.13 (p = 0.45)	-0.55 (p = 0.0004)
CM-RQ in males	0.41 (p = 0.094)	-0.14 (p = 0.68)	-0.43 (p = 0.11)
CM-RQ in females	-0.25 (p = 0.23)	-0.19 (p = 0.29)	-0.33 (p = 0.074)

Significance level: the p-value is indicated in parenthesis

### Analysis of variance

Results from the different models used are shown in Table 4. The line by sex interaction was always highly significant (p<0.001) for comb mass, but was moderately significant (p<0.05) or not significant for comb size. The line effect depended on the sex: the 3 lines differed significantly from each other for comb size and comb mass of males, whereas in females, the CH1 and NOE lines did not differ from each other and differed significantly from the WL-DJ line for comb size only.

**Table 4:** Significance levels for the different sources of variation in the analysis of comb size (CS) and comb mass (CM), according to the data set, with or without the RQ covariate.

Data set	R^2^	line	sex	line x sex	body weight	RQ
All data						
CM (n = 134)	0.766	p<0.0001	p<0.0001	p<0.0001	p<0.1126	p<0.5181
CS (n = 156)	0.826	p<0.0001	p<0.0001	p<0.0245	p<0.1624	p<0.0121
All data						
CM (n = 134)	0.765	p<0.0001	p<0.0001	p<0.0001	p<0.0928	-
CS (n = 156)	0.819	p<0.0001	p<0.0001	p<0.0245	p<0.1414	-
WL-DJ + NOE lines						
CM (n = 91)	0.744	p<0.0001	p<0.0001	p<0.0001	p<0.0548	p<0.2375
CS (n = 100)	0.788	p<0.0001	p<0.0001	p<0.1756	p<0.1593	p<0.0056
WL-DJ + NOE lines						
CM (n = 91)	0.739	p<0.0001	p<0.0001	p<0.0001	p<0.0420	-
CS (n = 100)	0.767	p<0.0001	p<0.0001	p<0.0381	p<0.1749	-
Heterozygous						
carriers of Pea-comb						
CM (n = 49)	0.825	p<0.0001	p<0.0001	p<0.0001	p<0.0792	p<0.0960
CS (n = 58)	0.817	p<0.0001	p<0.0005	p<0.1238	p<0.5497	p<0.0032
Heterozygous						
carriers of Pea-comb						
CM (n = 49)	0.814	p<0.0001	p<0.0001	p<0.0001	p<0.0454	-
CS (n = 58)	0.783	p<0.0001	p<0.0005	p<0.0294	p<0.4643	-
Line CH1						
CM (n = 43)	0.867	-	p<0.0001	-	p<0.2025	p<0.1390
CS (n = 56)	0.905	-	p<0.0001		p<0.6203	p<0.2122

The number of observations used in each analysis is indicated in parenthesis for each variable.

The WL-DJ line showed the largest and heaviest combs, whereas comb size was smallest in the NOE line, with the CH1 line being intermediate (Table 5). Body weight had no significant influence on comb size, whereas it showed an influence on comb mass when the analysis did not include line CH1 and omitted RQ (p<0.05). The RQ covariate had a significant effect on comb size but not on comb mass (Table 4). The level of significance of the RQ covariate on comb size increased when the analysis was performed on the subset of data from only the WL-DJ and NOE lines, and increased again when the analysis was restricted to the heterozygous carriers of the Pea-comb mutation in these two lines. Although the p-value of the RQ covariate for comb mass decreased in the data set of heterozygous carriers, it remained above the threshold of 0.05.

**Table 5:** Least squares means and standard error of the means for comb size, comb mass, body weight, and number of repeats (RQ) per line and sex.

	CH1	WL-DJ	NOE
Comb size of males	5301±166 ^b^ *	6739±198^a^	4312±178 ^c^
Comb size of females	1629±131^e^	2755±131^d^	1557±132 ^e^
Comb mass (g) of males	14.0±0.978 ^b^	24.5±1.06 ^a^	8.95±1.00 ^c^
Comb mass (g) of females	0.746±0.827 ^d^	2.72±0.74 ^d^	0.658±0.785 ^d^

*for a given trait, the least squares means showing a different superscript differ at p≤0.01

**Fig 6:**
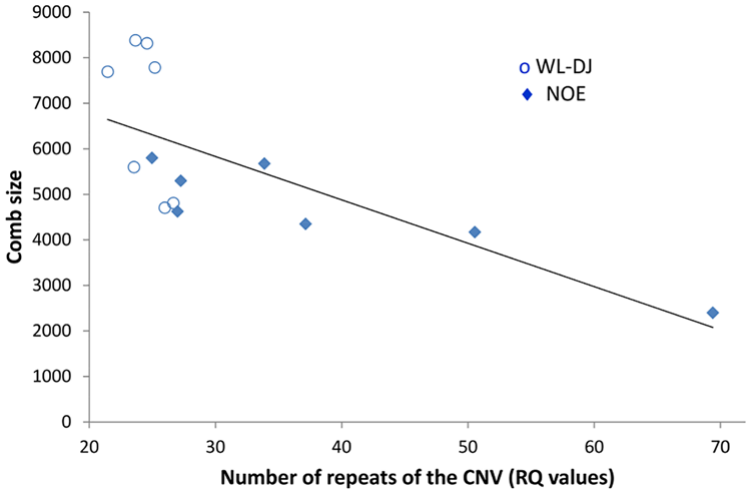
Variation of comb size as a function of the number of repeats (RQ values) for male heterozygous carriers of the Pea-comb mutation in the WL-DJ and NOE lines.

The linear regression coefficient of comb size on RQ was negative: it was-13.7 in the analysis of all data, -19.8 in the analysis of the WL-DJ and NOE lines, and-45.57 in the analysis restricted to the heterozygous carriers of Pea-comb. The negative relationship between comb size and RQ values is illustrated on Fig. 6 for male heterozygous carriers of Pea-comb, in the WL-DJ and NOE lines. The proportion of comb size variance explained by the model decreased when the RQ covariate was omitted: the largest loss of variance was observed in the analysis restricted to the heterozygous carriers and represented 3.4% of the total phenotypic variance. In the CH1 line, where all birds are homozygous carriers of Pea-comb, the effect of RQ as a covariate was neither significant for comb size (p = 0.21) nor for comb mass (p = 0.14).

## Discussion

### Line and sex effects

The importance of line and sex effects on size and mass of the normal single-comb has been known for a long time [13]. The sex effect always explained the largest proportion of the variance of comb size or comb mass in the present analysis, which is consistent with the fact that comb is a sexual ornament and that different quantitative trait loci were identified for comb size according to sex [9]. Regarding the line effect, the largest combs have generally been described in the White Leghorn, the breed of origin of the WL-DJ line [13], and rather high heritability values (0.5 to 0.6) were reported for comb size and comb mass in White Leghorn chickens [14]. This breed has been selected for egg production for many generations, which could have influenced hormonal regulations and affected comb size. The CH1 and NOE lines are unique experimental populations maintained at INRA which are not related to any other genotype previously studied for comb size.

Given that the Pea-comb mutation decreases the size of the comb, and considering that the relationship between mean and variance of a quantitative trait is generally positive, the large variation of comb size observed in this study was rather unexpected. The variation of comb mass was even greater, suggesting that comb mass may be influenced by a larger number of factors, including body weight. The lower correlation between comb mass and comb size that was observed in females as compared to males could be explained by 3 factors: (i) the 2D picture was more difficult to obtain in females since combs were small and often fell to one side, so that their surface was more difficult to estimate from a picture, (ii) a larger variation of conformation of the comb was suggested in females by the 2D morphometry [15] (iii) comb mass was measured 2 months after the picture for comb size was taken, and some hens may have stopped laying and undergone hormonal changes. Furthermore, the adult body weight influenced the variation of comb mass but not that of comb size, which may be particularly important in WL-DJ females, because the sex-linked dwarf gene was segregating in this line.

The distribution of RQ values differed between heterozygous and homozygous Pea-comb carriers and it could be possible to infer the zygosity at the Pea-comb locus on the basis of the number of repeats. However, it is not possible to propose an absolute rule because of line and sex effects. Thus, a within-line training data set would always be necessary in order to use RQ values to infer Pea-comb genotype.

### Effect of the CNV on comb measurements

The number of repeats had a significant effect on comb size which could be approximated by a linear regression. The regression coefficient was negative, corresponding to the fact that the Pea-Comb mutation has a negative effect on comb size: the higher the number of repeats, the higher the reduction in comb size. The contribution of the CNV to 3.4% of the phenotypic variance of comb size in heterozygous carriers of the Pea-comb mutation, corresponds to a locus effect of 0.185 σ_p_; in comparison, commonly detected QTL effects were found to vary from 0.2 to 1 σ_p_ in dairy cattle [16] and the authors of this meta-analysis considered that a large number of QTL with smaller effects (<0.2 σ_p_) went undetected because of the lack of statistical power of QTL detection studies. Thus, the *SOX5*-CNV locus in Pea-comb chickens may be considered to be a QTL of comb size with a small but significant effect. The *SOX5* gene lies within one of the QTL region previously identified for the variation of comb mass in wild-type females [9], but this QTL region was rather large and the gene, or genes, underlying this QTL have not been determined. Furthermore, the linkage disequilibrium has not yet been characterized in the experimental lines used for the present study, so it is not possible to make any inference regarding the QTL region in these lines. The negative phenotypic correlation between comb size and RQ values in females, clearly significant in the NOE line, could support the hypothesis that *SOX5* may actually be involved in this QTL, considering that some quantitative variation of the number of repeats can take place in wild-type birds [8]. Yet, the negative linear relationship found in heterozygous carriers between RQ values and comb size was also found in males, so that the effect of *SOX5* cannot solely be restricted to comb size of females, as was the case for the QTL.

The effect of the CNV was observed on comb but not on comb mass although these two traits are positively correlated. There was a slightly lower number of observations for comb mass, which decreases the power of the statistical analysis. In general, the proportion of variance explained by a given statistical model was slightly lower for comb mass than for comb size, which suggests that other factors not included in the model may influence comb mass and not comb size. The possible explanations previously given for the lower correlation between comb size and comb mass found in females as compared to males, could also hold here and explain why the quantitative effect of the CNV was more easily observed on comb size than on comb mass.

The fact that the linear relationship between CNV and comb size was significant in heterozygous carriers but not in homozygous carriers may be due to line effects, since homozygous carriers could only be studied in the CH1 line, and line was shown to greatly influence comb size. However, a line effect would not explain the negative relationship between RQ values and comb size obtained in heterozygous carriers of both the WL-DJ and NOE lines. Such a negative relationship suggests an increasing impact of the *SOX5*-CNV on comb size when the number of repeats is increasing on a single chromosome, whereas this impact may have reached its maximum in homozygous carriers of the CNV. Since the Pea-comb mutation has been shown to decrease the Sonic hedgehog receptor expression [10], one could understand that there is a limit to this effect when expression of down-stream effectors may reach a minimal value. This would mean that a higher number of tandem repeats would increase the inhibitory action on comb morphogenesis due to the ectopic expression of *SOX5*, towards a maximum value when both chromosomes show a high number of repeats. Indeed, some heterozygous carriers in the NOE line showed a number of repeats as high as the total number of repeats of homozygous carriers in the CH1 line, and they exhibited the smallest combs of their line. The underlying mechanism could involve a change in chromatin conformation disturbing the action of regulatory elements and leading to the ectopic expression pattern of *SOX5*, as previously suggested [8].

## Conclusions

This study shows the quantitative effect of a CNV on a continuous trait measured in adult animals. The CNV of *SOX5* is an expansion of a pre-existing CNV in a non-coding region. The present results suggest that heterozygosity for such a CNV may contribute to phenotypic plasticity, i.e to phenotypic variation within a given genotype (Pea-comb). Such a phenomenon could contribute to the better fitness generally associated with heterozygosity and genetic diversity. Although this is observed here for a morphological trait, other examples should be identified in order to investigate the correlation between CNVs in non-coding regions and a quantitative trait, in the absence of any deleterious health effect.

## Supporting Information

S1 File The full text, in French, with tables and figures, corresponding to reference #15. (PDF)

S1 Table The whole data set with animal identification, phenotypes and genotypes for both sexes in the three lines. (XLSX)
