# Supplementary material for: Quantitative Effect of a CNV on a Morphological Trait in Chickens
Source: PLoS One. 2015 Mar 13;10(3):e0118706. doi: 10.1371/journal.pone.0118706 (PMC4358827; doi:10.1371/journal.pone.0118706)

PHÉNOTYPAGE ET FACTEURS DE VARIATION DE LA TAILLE DE LA CRÊTE : APPLICATION À LA CRÊTE EN POIS

Moro Céline[1], Cornette Raphaël[2], Vieaud Agathe[1], Gourichon David[3], Bed'hom Bertrand[1], Tixier-Boichard Michèle[1]

[1]*INRA, UMR1313 Génétique Animale et Biologie Intégrative, F-78352 JOUY-EN-JOSAS, France*
[2]*MNHN Plate-forme de morphométrie, 45 rue Buffon, F-75005 PARIS, France*
[3]*INRA, UE1295 Pôle d'expérimentation avicole de Tours, F-37380 NOUZILLY, France*

michele.boichard@jouy.inra.fr

RESUME

La taille de la crête présente une importante variabilité génétique chez le poulet. La mutation crête en pois *P* a une taille réduite et une forme modifiée. Cette étude présente une approche de morphométrie géométrique à 2 dimensions pour déterminer la taille et la conformation de la crête en pois. La crête de coqs et poules adultes, de 3 lignées expérimentales de type ponte, a été photographiée (178 animaux) et pesée après abattage (150 animaux). L'analyse des photographies a permis de définir 150 points repère (landmarks). L'analyse générale Procruste calcule la taille centroïde (= racine carrée de la somme des distances entre les landmarks et le centre de gravité de l'objet). Les coordonnées des landmarks obtenues par l'analyse Procruste donnent la conformation de chaque crête. Les effets lignée, sexe et leur interaction expliquent 83% de la variance de la taille centroïde. L'effet lignée est plus important chez les mâles que chez les femelles. Les corrélations entre taille centroide et masse de la crête étaient toujours significatives mais plus élevées chez les mâles (0,85 à 0,96 selon la lignée) que chez les femelles (0,61 à 0,82). La taille centroide apparait comme un meilleur prédicteur de la masse de la crête que le poids corporel. La conformation des crêtes était plus variable chez les femelles que chez les mâles. La prise de photographies est plus délicate chez les femelles dont la crête est petite et tombante. En conclusion, l'analyse morphométrique permet de quantifier de manière fiable la taille et la conformation de la crête *in vivo* mais une analyse en 3D permettrait de tenir compte des variations d'épaisseur de la crête.

ABSTRACT
Phenotyping and factors of variation of comb size: application to the Pea-comb

Comb size exhibits a large genetic variability in chickens. The Pea-comb mutation shows a reduced size and a modified shape. This study presents an approach of geometric morphometry to estimate the size and conformation of the Pea-comb. 178 adult males and females were sampled in 3 experimental egg-type lines. Pictures were taken for each comb side, adult body weight was measured and a subset of 150 animals was slaughtered to measure comb mass. The 2-dimensions analysis of the pictures was based upon 150 landmarks for each comb. The Procruste analysis calculated the centroid size as the square root of the sum of the distances between landmarks and the weighted center of the comb. The landmark's coordinates obtained from the Procruste analysis provided the conformation of each comb. The main factors of line, sex and line x sex interaction explained 83% of the variation of the centroid size. The line effect was larger in males than in females. Phenotypic correlations between the centroid size and comb mass were all significant but were higher in males (0.85 to 0.96) than in females (0.61 to 0.82). Centroid size was found to be a better predictor of comb mass than body weight. Comb shape was more variable in females than in males. Taking pictures of the comb was more difficult in females whose comb is smaller and more or less falling on one side. In conclusion, the 2D-morphometric analysis succeeded in providing a reliable quantified estimate of comb size and comb shape *in vivo*, but a 3D analysis would be useful to take into account variations in comb width.

INTRODUCTION

La crête est un appendice présentant un fort dimorphisme sexuel qui influence le rang social des animaux mais aussi l'adaptation à la température. La taille de la crête a été étudiée en tant que mesure quantitative de l'activité des hormones androgéniques ou comme indicateur de capacité reproductive. Les mesures de longueur L et hauteur H sont simples et peuvent être effectuées sur animaux vivants, contrairement au poids de la crête, qui nécessite de tuer l'animal, mais permet d'étudier les variations de l'épaisseur non prise en compte par les mesures de longueur. La longueur est aussi la mesure quantitative la plus précise des réponses aux stimuli externes et internes. Le produit LxH a été proposé par Jones et Lamoreux (1943) comme le critère le plus corrélé à la masse de la crête, avec des valeurs variant de 0,845 à 0,952 selon l'origine, le type d'éclairage et l'âge des oiseaux étudiés. Une importante variabilité génétique de la taille de la crête a été décrite entre races et intra-race chez le poulet domestique, avec des valeurs d'héritabilité comprises entre 0,54 et 0,6 pour la taille ou le poids de la crête en race Leghorn blanche (Tufvesson et al., 1999). Plusieurs QTL ont été découverts pour la masse de la crête (Wright et al., 2008). Deux QTL situés sur le chromosome 1, dont l'un englobe le locus *P*, ne concernent que la masse de la crête femelle, ce qui suggère que l'architecture génétique de la crête est en partie distincte entre les 2 sexes. Plusieurs mutations modifient la forme de la crête, la mutation *P*, dominante, détermine le phénotype appelé crête en pois, caractérisé par une taille réduite, et une forme modifiée, plus compacte, avec trois rangées longitudinales de petites pointes, évoquant de loin la forme d'un pois. Cette mutation améliore légèrement l'efficacité alimentaire (Mérat et Bordas, 1979). L'objectif de ce travail est de tester une méthode d'analyse morphométrique afin d'étudier les facteurs de variation de la taille et de la forme de la crête en pois, pour laquelle les mesures simples de longueur et hauteur paraissent moins adaptées que pour la crête de forme normale. Cette méthode pourrait servir à quantifier d'autres paramètres morphologiques de façon non invasive chez le poulet.

1. MATERIELS ET METHODES

1.1. Animaux

Trois lignées expérimentales de type ponte ont été utilisées : la lignée 'Cheptel 1' (CH1) est homozygote pour la mutation *P* alors que la lignée 'Doubles Jaunes' (DJ : lignée White Leghorn sélectionnée pour le taux d'œufs doubles) et le troupeau Noé (population non sélectionnée porteuse de différentes mutations de plumage) comportent des animaux homozygotes ou hétérozygotes pour la mutation *P*. Selon leur ascendance et descendance, le génotype hétérozygote a pu être déterminé pour 20 mâles et 33 femelles, mais il n'existe pas de marqueur moléculaire permettant de diagnostiquer le génotype hétérozygote pour *P*. La mutation nanisme liée au sexe est en ségrégation dans la lignée DJ et affecte fortement le poids corporel. A l'âge de 120 jours, les poules sont mises en cage individuelle pour la ponte et les coqs sont 1 à 2 par cage, dans une cellule différente. La température ambiante était de 21°C, les poules recevaient 14 h et les mâles 12 h de lumière par jour. Les animaux sont nourris *ad libitum*. L'effectif total d'animaux porteurs de *P* était de 179 (Tableau 1).

1.2. Mesures

Les animaux ont été pesés et photographiés à 300 jours d'âge, à l'aide d'un Nikon Coolpix L110 en mode Macro avec un objectif Nikkor 15X (Optical Zoom Wide VR 5.0-75.0 mm 1 :3.5-5.4), au même zoom (X6) et à la même échelle, avec une distance objectif-plan de 27 cm. Les deux profils des animaux ont été photographiés afin de choisir la photo où la crête était la plus droite possible et où la surface de la crête était maximisée. A 390 jours d'âge, les animaux ont été sacrifiés par électronarcose, décapités, puis les crêtes de 94 femelles et 56 mâles (certains étant conservés pour d'autres études) ont été découpées à l'aide d'un scalpel (de lame 23), parfois nettoyées et pesées à 0,1g près et conservées à -20°C.

1.3. Analyse morphométrique

L'analyse de morphométrie géométrique utilise les coordonnées de points repères (ou *landmark*) pour quantifier la forme de la crête et séparer les composantes 'taille' et 'conformation'. La méthode des *landmarks* et l'analyse TPS (*Thin Plate Splines*) décrivent l'objet grâce à des points homologues dont la position varie en fonction de la taille et la conformation de chaque spécimen. Avec le logiciel tpsDig2, 150 *landmarks* ont été définis pour décrire la courbe de la crête sur chaque photographie. Les *landmarks* anatomiques sont les 2 points repères qui marquent le début et la fin de la crête (Figure 1). Le processus de superposition Procruste ramène toutes les mesures sur une même échelle afin d'analyser la conformation indépendamment des différences de taille, selon trois étapes (Figure 2) :

- la translation des objets par centrage : les objets sont superposés par leur centre de gravité
- la normalisation : tous les objets sont mis à la même échelle
- la rotation d'un spécimen par rapport à un autre ou de l'ensemble des spécimens par rapport à un objet consensus : l'angle de rotation dépend du critère de rotation choisi, ici celui des moindres carrés qui vise à minimiser l'ensemble des distances entre les points repères homologues pour deux objets.

L'analyse générale par le logiciel tpsRelw a permis d'obtenir la taille centroïde et de comparer les

conformations de chaque spécimen. La taille centroïde est la racine carré de la somme des distances entre les *landmarks* et le centre de gravité de chaque objet, c'est une mesure de la taille non-corrélée à la conformation. Les coordonnées des *landmarks* (ou encore résidus de l'analyse Procruste) sont utilisées pour décrire la conformation de chaque objet, et les rendre comparables (Mitteroecker et Gunz 2009).

1.4. Analyse statistique

Un premier modèle d'analyse de variance a été appliqué à toutes les données de taille centroide (TC) et de masse de la crête pour estimer les effets de la lignée, du sexe et de l'interaction lignée x sexe, avec le poids adulte en covariable (modèle 1). Un deuxième modèle a été appliqué séparément sur chaque sexe pour estimer les effets de la lignée et de la famille paternelle intra-lignée, pour les variables TC et masse de la crête, avec le poids adulte en covariable (modèle 2). Les corrélations phénotypiques entre les variables TC, masse de la crête et poids adulte ont été estimées par lignée et par sexe. Concernant la conformation, une analyse en composantes principales a été réalisée sur les coordonnées des *landmarks* issues du processus Procruste. Un graphe d'allométrie a été produit pour visualiser les relations entre la conformation de la crête et la TC en fonction de la lignée et du sexe.

2. RESULTATS

2.1. Facteurs de variation des mesures de la crête

Le modèle 1 explique 83% de la variance totale pour la TC et 77% pour la masse de la crête. L'interaction entre lignée et sexe est significative et s'explique par le fait que les 3 lignées diffèrent pour la TC des mâles alors que seule la lignée DJ diffère des 2 autres pour la TC des femelles. La régression sur le poids corporel est proche de la signification pour la masse de la crête ($p<0{,}0679$). A poids adulte constant, la lignée DJ présente les crêtes les plus grandes et les plus lourdes pour les deux sexes, alors qu'elle est aussi la lignée la plus légère (Tableau 2). A l'inverse, la lignée Noé présente les animaux les plus lourds et les crêtes les plus petites. Le modèle 2 explique de 74% à 78% de la variance chez les mâles et 76 à 80% chez les femelles. L'effet lignée et l'effet père intra-lignée sont significatifs sur les caractères TC et masse de la crête pour chaque sexe ; la régression sur le poids corporel n'est pas significative chez les mâles mais est significative pour la masse de la crête des femelles.

2.2 Corrélations phénotypiques

Les corrélations entre TC et masse de la crête sont toujours significatives mais sont plus élevées chez les mâles que chez les femelles, la différence entre sexes était la plus grande dans le cheptel 1 (Tableau 3). Les corrélations entre TC et masse de la crête sont toujours supérieures aux corrélations entre masse de la crête et poids corporel, la variable TC est donc un meilleur prédicteur de la masse de la crête que le poids corporel. Les corrélations entre TC et poids corporel ne sont pas significatives chez les mâles des 3 lignées et les femelles du troupeau Noé, et sont inférieures à 0,5 chez les femelles DJ et CH1.

2.3 Relation entre conformation et taille centroide

Le graphe d'allométrie entre la taille centroïde et la conformation des crêtes (Figure 3) montre une plus grande variabilité de conformation des crêtes chez les femelles que les mâles, alors que celles-ci présentent des tailles de crête inférieurs à celles des mâles. Les femelles du cheptel 1 et du troupeau Noé ont une conformation des crêtes assez similaire alors que les femelles DJ ont une conformation différente, plus proche de celle de la crête des mâles.

3. DISCUSSION

3.1 Effets de la lignée, du sexe et du poids corporel

Des différences notables de la taille de la crête sont connues entre races : les poules de race Leghorn Blanche présentaient une longueur et une aire de crête significativement plus grandes que celles des races Rhode Island Rouge et Barnevelder (Axelsson 1933). La lignée White Leghorn DJ étant aussi sélectionnée sur le taux d'ovulations multiples, ce caractère pourrait être associé à des modifications hormonales susceptibles d'affecter la crête, mais aucune corrélation significative n'a été obtenue entre la taille ou la masse de la crête et le taux individuel d'œufs doubles (données non montrées). Le poids corporel n'a un effet significatif que chez les femelles de la lignée DJ, ce qui s'explique par la coexistence de poules naines et normales dans cette lignée.

L'effet du sexe sur la taille et la masse de la crête coïncide bien avec le dimorphisme sexuel observé sur les caractères secondaires chez de nombreuses espèces, ainsi qu'avec la découverte récente de différents QTL de masse de la crête pour le coq et la poule (Wright *et al.,* 2008). La visualisation des conformations moyennes des crêtes en fonction du sexe a montré que la crête des mâles a une forme qui va plus loin vers l'arrière de la tête (sans être attachée à la base). De plus, la conformation des crêtes des individus homozygotes pour *P* (CH1) semble être moins variable. Bien que les femelles présentent des tailles et masses de crête inférieures à celles des mâles, elles présentent une plus grande variabilité de conformation. Plusieurs QTL sont décrits pour la masse de la crête femelle et un seul est commun aux deux sexes, ces QTL pourraient influencer la variabilité de conformation de la crête chez les femelles.

3.2 Prédiction de la masse de la crête

L'objectif est de contribuer au phénotypage d'un appendice de dissipation de chaleur et d'analyser plus finement l'effet de la mutation P sur la morphogénèse de la crête. Les corrélations phénotypiques entre TC et masse permettent de proposer la taille centroïde comme une mesure fiable de la masse de la crête, non invasive et réalisable à différents âges. L'analyse d'images en 2 dimensions est assez simple même si elle demande un peu de temps, et les logiciels d'analyse sont libres. Cependant, des corrélations plus faibles entre TC et masse sont observées chez les femelles, ce qui peut s'expliquer par plusieurs facteurs : (1) les mesures de masse ont été effectuées plus tard que la prise de photographies, il est possible que certaines poules n'aient plus été en ponte à ce moment-là, ce qui est associé à une régression de leur crête (2) la prise de photographies est plus difficile chez les poules dont la crête est souvent tombante et l'erreur de mesure de la taille peut être plus grande (3) la conformation de la crête des femelles est plus variable que celle des mâles ce qui peut limiter l'efficacité d'une analyse 2D qui ne tient pas compte de la largeur de la crête, dimension plus variable et plus importante chez les crêtes en pois que chez les crêtes simples, où la simple lame a une largeur peu variable. Chez les mâles, la corrélation était la plus faible dans la lignée DJ, ce qui peut être du à la plus grande taille de ces crêtes.

CONCLUSIONS

L'analyse morphométrique a l'avantage de permettre de quantifier la taille et la conformation des crêtes de façon indépendante. Elle représente une approche intéressante pour le phénotypage de caractères de morphologie chez la poule. Les formes complexes et variées des crêtes en pois semblent nécessiter une mesure de volume pour avoir une estimation complète de la forme de la crête chez les femelles, mais aussi chez les mâles dans le cas de lignées ayant de très grandes crêtes, comme la Leghorn blanche.

REFERENCES BIBLIOGRAPHIQUES

Axelsson, J., 1933. Proc 5th World's Poultry Congress, Rome, (Italie) 2 : 273-296.
Jones, D.G., Lamoreux, W.F., 1943. Endocrinol., 32 : 356-260.
Mérat, P., Bordas, A., 1979. Brit. Poult. Sci., 20 : 463-472.
Mitteroecker, P., Gunz, P., 2009. Evol. Biol., 36 : 235-247.
Tufvesson, M., Tufvesson, B., Von Schantz, T., Johansson, K., Wilhelmson, M., 1999. J. Anim. Breed. Genet., 116 : 127-138.
Wright, D., Kerje, S., Brändström, H., Schütz K., Kindmark, A ., Andersonn, L., Jensen, P., Pizzari, T., 2008. Evolution, 62 : 86-98.

Tableau 1. Effectifs d'animaux étudiés par caractère, par lignée et par sexe

Caractère	CH1 mâles/femelles	DJ mâles/femelles	Noé mâles/femelles	Total
Taille centroide	24/39	17/39	21/39	179
Masse de la crête	20/28	17/35	19/31	150
Poids adulte	23/39	17/39	21/39	178

Tableau 2. Moyennes et erreur standard de la moyenne par caractère, lignée et sexe

	Cheptel 1	Lignée DJ	Troupeau Noé
Taille centroide des mâles	5302±167	6739±381	4312±244
Taille centroide des femelles	1629±62,9	2755±120	1540±72,5
Masse de la crête (g) des mâles	14,0±0,968	24,5±2,61	8,95±1,05
Masse de la crête (g) des femelles	0,746±0,037	2,72±0,205	0,658±0,058
Poids adulte (g) des mâles	2646±44,6	2234±96	3180±125
Poids adulte (g) des femelles	1977±28,7	1799±57,5	2331±56,8

Tableau 3. Corrélations phénotypiques entre les mesures de crête par lignée et sexe

	Cheptel 1	Lignée DJ	Troupeau Noé
Entre TC et masse de la crête des mâles	0,92	0,85	0,96
Entre TC et masse de la crête des femelles	0,61	0,82	0,75
Entre poids corporel et masse de la crête			
des mâles	NS	0,46	NS
des femelles	0,52	0,57	0,58

Figure 1: Profils droits de deux coqs (A) et (B) et une poule (C) ayant la crête en pois. Les *landmarks* forment le contour de la crête. Les *landmarks* « anatomiques », signalés par les flèches, indiquent le début et la fin de la crête.

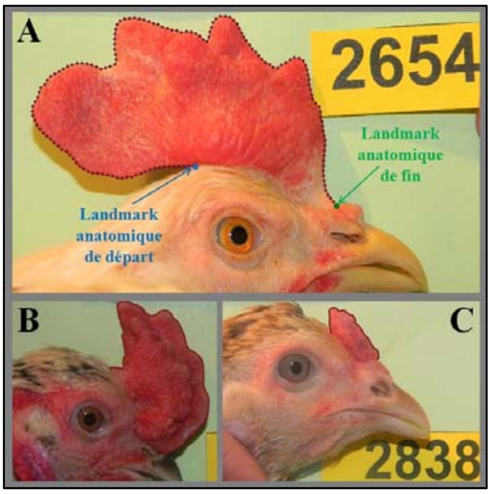

Figure 2: Processus de superposition Procruste pour comparer la forme des crêtes indépendamment de leur taille

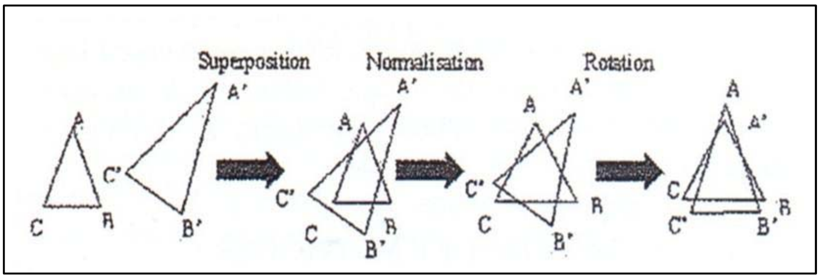

Figure 3: Relation entre la TC et la coordonnée sur l'axe 1 de l'analyse en composantes principales (ACP) des conformations des crêtes, en fonction du sexe (M=mâle ; F=femelle) et des lignées (code couleur sur la figure)

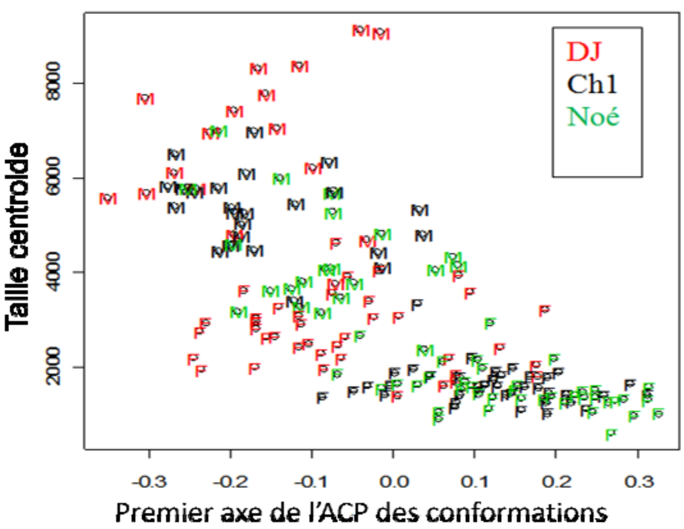

Supplement: S1 File — (PDF) [file pone.0118706.s001.pdf]
